# Phytochemical investigation and antimicrobial appraisal of *Parrotiopsis jacquemontiana (Decne)* Rehder

**DOI:** 10.1186/s12906-018-2114-z

**Published:** 2018-01-31

**Authors:** Saima Ali, Muhammad Rashid Khan, Irfanullah, Moniba Sajid, Zartash Zahra

**Affiliations:** Department of Biochemistry, Faculty of Biological Sciences, Quaid-i-Azam University, Islamabad, 45320 Pakistan

**Keywords:** *Parrotiopsis jacquemontiana*, Multi drug resistance, Minimum inhibitory concentration, Bacterial strains, Fungal strains

## Abstract

**Background:**

*Parrotiopsis jacquemontiana (Decne)* Rehder. is locally used for skin infections and in wound healing. In this study we have evaluated methanol extract of its leaves and derived fractions against the clinical multi-drug resistant bacterial strains.

**Methods:**

*P. jacquemontiana* leaves powder extracted with 95% methanol (PJM) and fractionated in escalating polarity of solvents; n-hexane (PJH), chloroform (PJC), ethyl acetate (PJE), n-butanol (PJB) and the remaining as aqueous fraction (PJA). Clinical as well as environmental 19 bacterial strains and 8 fungal strains were screened for minimum inhibitory concentration (MIC) and minimum bactericidal/fungicidal concentration (MBC/MFC). Preliminary phytochemical investigation for various phytochemical classes was also carried out.

**Results:**

PJM contained the coumarins, phenols, flavonoids, tannins, alkaloids, glycosides, saponins, sterols, phlobatannins, steroids, phytosterols, triterpenoids, acids, quinones, proteins, vitamin C, betacyanins, oils and resins while anthraquinones, phytosteroids, carbohydrates and anthocyanins were not detected. Disc diffusion assay (1 mg/disc) indicated the sensitivity of all the MDR strains of bacteria with PJM, PJE and PJB, while no inhibition was recorded with PJA. PJH and PJC inhibited the growth of all the strains of *Staphylococcus aureus*, *Pseudomonas aeruginosa* and *Coagulase negative staphylococci* used in this study*.* Maximum zone of inhibition (35.5 ± 1.32 mm) was obtained with PJM against *Staphylococcus lugdenesis* MDR (6197). Comparatively lower MIC (8-64 μg/ml) and MBC (32-256 μg/ml) values were recorded for PJM and PJE. In case of fungal strains only PJM, PJE and PJB markedly inhibited the growth and lower MIC (8-128 μg/ml) and MFC (32-512 μg/ml) values were determined for PJM and PJE.

**Conclusion:**

The remarkable inhibition of various bacterial and fungal strains at low doses of the extract/fractions suggested the strong antibacterial, antifungal and anti-candidal potential of *P. jacquemontiana* leaves.

## Background

Infectious diseases represent one of the major health concerns and are the 2nd major cause of loss of productive life and death worldwide. These also remain as one of the leading cause of loss of productive life worldwide. Among the death caused by microorganisms 70% are caused by bacterial infections [1].

A number of important antibiotics including tetracyclines, macrolides, cephalosporins and aminoglycosides have been developed for solving major problems of infectious diseases. However, these valuable compounds are now facing a risk of losing their worth because multiple drug resistance has been advanced in plant and human pathogenic microorganisms [2–4]. The global emergence of several microorganisms such as *Klebsiella pneumoniae, Haemophilus* and *Escherichia coli* and a variety of β-lactamase producers are accounting for major therapeutic problems nowadays. These multi-drug resistant strains of *K. pneumoniae* and *E. coli* are found abundantly in hospitals and isolated from acquired community infections [4, 5]. Methicillin-resistant *Staphylococcus aureus* (MRSA) is not only impervious to methicillin (originally designed to eradicate penicillinase producing *S. aureus*) but also to other antibiotics such as chloramphenicol, tertracycline, aminoglycosides and lincosamides etc. Increasing resistance in these strains is also observed against disinfectants which is a leading cause of clinically-acquired infections [6], therefore, there is a need to explore plant based therapeutic agents that are effective against MRSA [7, 8]. *Candida albicans* is also reported to be responsible for causing invasive candidiasis contributing to about 50-70% of the cases [9]. This present condition has compelled scientists to evaluate novel antimicrobial agents from different sources including medicinal plants [10, 11]. Antimicrobial testing can be used for discovery of new drugs, epidemiology and their possible therapeutic outcome [12].

One of the known plants reported for its aromatic and medicinal properties is *Parrotiopsis jacquemontiana (Decne)* Rehder. It is a commonly distributed wild shrub/small tree of about 5 m tall residing in the forests of Upper Dir, Pakistan. Its vernacular name is Beranj and belongs to the family Hamamelidaceae [13]. Leaves of this plant are reported to have wide use for combatting skin infections, skin eruptions and for treating general body pain [14, 15]. The leaves are crushed and placed 2-3 days on the wounded area for healing. Oil can be extracted from its stem and placed on the affected area [16]. Up till now, no literature data was found relevant to its biological activities including antibacterial, antifungal, and phytochemical investigation. Therefore, the main objectives of the present study conducted were to evaluate the antibacterial (clinical multidrug resistant strains; MDR), antifungal and phytochemical constituents of the methanol extract of *P. jacquemontiana* and its derived fractions.

## Methods

### Collection and identification of plant material

The leaves of *P. jacquemontiana* were collected from the forests of Upper Dir, Pakistan in the month of May-June 2016. It was recognized by its local name and then taxonomically identified and confirmed by a senior plant taxonomist Syed Afzal Shah of Plant Sciences Department, Quaid-i-Azam University, Islamabad, Pakistan. The voucher specimen (063214) was deposited at the Herbarium of Pakistan, Quaid-i-Azam University, Islamabad, Pakistan.

### Preparation of plant material and fractionation

The collected leaves were cleaned to remove from dust particles and then placed under shade drying for about two to three weeks. The dried plant sample was then ground to a fine texture powder by subjecting it to a 60-mesh size Willy mill. About 1 kg of the plant powder was extracted with 5 l of 95% methanol (thrice) for 72 h and then filtered with Whatman No. 1 filter paper. The filtrate was combined and concentrated on a rotary evaporator under reduced pressure at 40 °C. For fractionation purpose, a weighed (50 g) portion of the extract (PJM) was suspended in distilled water (400 ml) and exposed to liquid-liquid partition by using different solvents (400 ml) in order to resolve the compounds with escalating polarity. The fractionation by each solvent was carried out thrice. The solvents were used in the order of n-hexane (PJH), chloroform (PJC), ethyl acetate (PJE), and n-butanol (PJB) while the soluble residual aqueous material was termed aqueous fraction (PJA). The respective solvents were again evaporated with rotary evaporator under reduced pressure. The fractions were dried, collected, weighed and stored at 4 °C until phytochemical and antimicrobial activities were performed.

### Screening of phytochemical constituents

Phytochemical components of PJM and its derived fractions were analyzed qualitatively by using different standardized test procedures [17–21].

#### Alkaloid detection

##### Mayer’s test

According to this test procedure, 2 ml of concentrated HCl was added to 2 ml of the respective plant extract samples followed by an addition of few drops of Mayer’s reagent. Either formation of white precipitate or green color confirmed the existence of alkaloids in that tested sample.

##### Hager’s test

For this test procedure, few drops of Hager’s reagent (saturated picric acid solution) added to 2 ml of the respective plant extract. Bright yellow precipitate formation indicated the existence of alkaloids [17].

#### Anthocyanin and betacyanin detection

An aliquot of 1 ml of 2N NAOH was added to 2 ml of each plant extract and heated at 100 °C for about 5 min to assess the presence of anthocyanin and betacyanin. Bluish green color formation specified the existence of anthocyanin while yellow color specified the existence of betacyanin in the plant samples tested [18].

#### Anthraquinone detection

According to [19] few drops of 2% HCl were added to the tested plant extracts. Red precipitate formation indicated the existence of anthraquinones in the samples, while another test procedure [17] involved the addition of 1 ml benzene to 1 ml of test sample followed by the addition of 10% ammonia solution. Red color formation indicated the existence of anthraquinones in the samples.

#### Coumarins detection

A volume of 1 ml of 10% NAOH solution was added to 1 ml of the plant samples. Yellow color formation confirmed the existence of coumarins in the tested samples [19].

#### Flavonoids detection

##### Alkaline reagent test

According to [18], 1 ml of 2N NAOH solution was added to 1 ml of plant extract samples. Yellow color appearance indicated the existence of flavonoids in the sample.

##### FeCl_3_ test

Few drops of FeCl_3_ solution was added to 1 ml of plant extract samples. Blackish red precipitate revealed the existence of flavonoids in the test samples [17].

#### Glycosides detection

##### Keller Killani test

A volume of 1 ml of glacial acetic acid was added to 1 ml of plant extract samples and cooled. After cooling, 2 drops of FeCl_3_ was added followed by careful addition of conc.H_2_SO_4_ along the walls of the test tube. Reddish brown colour ring formed at the junction of two layers indicated the existence of glycosides [17].

#### Saponin detection

According to [19], 2 ml of distilled water was added to 2 ml of plant extract sample and shaken vigorously lengthwise for 15 min in a graduated cylinder. A layer of foam 1 cm or more thick confirmed the existence of saponins in the test samples.

#### Tannin detection

##### FeCl_3_ test

A volume of 2 ml of 5% FeCl_3_ was added to 1 ml of plant extract samples. Appearance of greenish black or dark blue color confirmed the existence of tannins in the test samples [18].

##### Alkaline reagent test

A volume of 2 ml of 1N of NaOH was added to 2 ml of plant extract samples. Appearance of yellow to red color revealed the existence of tannins [17].

##### Bromine water test

Plant extracts were dissolved in 50% alcohol and filtered. The filtrate was reacted with 3-4 drops of bromine water. Appearance of buff color indicated the existence of condensed tannins in the plant test sample whereas hydrolysable tannins gave no such indication [20].

#### Terpenoid detection

An aliquot of 1 ml of 1% HCl was added to 2 ml of plant extract samples and left to stand for 5-6 h. Later on, 1 ml of Trim-Hill reagent was added to it and heated in a boiling water bath for 5-10 min. Appearance of bluish green color indicated the existence of terpenoids [17].

#### Phenol assessment

##### Ellagic acid test

Few drops of 5% glacial acetic acid were added to 1 ml of plant extract samples followed by addition of few drops of 5% NaNO_2_ solution. Muddy brown color formation revealed the existence of phenols in the test samples [17].

#### Sterol detection

##### Salkowski’s test

In this test procedure 5 ml of chloroform was added to 2 ml of plant extract samples followed by careful addition of 1 ml of conc.H_2_SO_4_ along the walls of the tube. Reddish brown color in the lower layer indicated the existence of sterols in the test samples [17].

#### Steroid and Phytosteroid detection

To 1 ml of plant extract sample, equal volume of chloroform was added. After subjecting the mixture to few drops of conc.H_2_SO_4,_ ring formation took place. Appearance of a brown color ring marked the existence of steroids whereas appearance of bluish-brown ring color marked the existence of phytosteroids in the test samples.

#### Phytosterol detection

##### Libermann-Buchard’s test

Plant extracts were filtered after treating with chloroform. The filtrate obtained was treated with few drops acetic anhydride, boiled and cooled at room temperature. Conc.H_2_SO_4_ was added and formation of brown-color ring at the junction demarcated the existence of phytosterols in the test samples.

#### Phlobatannins detection

To a volume of 1 ml plant extract sample, few drops of ammonia solution (10%) were added. Pink color precipitates designated the existence of Phlobatannins in test samples.

#### Triterpenoids detection

1 ml of Libermann-Buchard Reagent (acetic anhydride + conc.H_2_SO_4_) was added to 1.5 ml of plant extract samples. Appearance of bluish-green color marked the existence of triterpenoids in the test samples.

#### Quinones detection

1 ml of conc.H_2_SO_4_ was added to plant extract sample (1 ml). Red color appearance designated the existence of quinones in the test samples.

#### Acid detection

1 ml plant extract sample was treated with a solution of sodium bicarbonate. Effervescence formation indicated the existence of acids in the test samples.

#### Vitamin C detection

##### DNPH test

1 ml plant test solution was reacted with Dinitrophenyl hydrazine (dissolved in conc.H_2_SO_4_). Yellow precipitate formation marked the existence of vitamin C in the test samples.

#### Protein detection

##### Xanthoproteic test

The plant extract sample (1 ml) was treated with few drops concentrated Nitric acid according to this procedure test. Yellow color formation indicated the presence of proteins in the test samples.

##### Biuret test

NAOH solution (40%) was added in equal volume to 0.5 mg plant extract solution followed by addition of few drops CUSO_4_ solution (1%). Violet color appearance in the test samples marked the existence of proteins.

#### Carbohydrate detection

##### Benedict’s test

Few drops of Benedict’s reagent (alkaline solution of cupric-citrate complex) were mixed with test solution samples followed by boiling in water bath. Reddish-brown precipitate formation indicated the existence of carbohydrates in the test samples.

#### Oils and resins detection

##### Filter paper test

Plant test samples were applied on filter paper. The development of transparent appearance on filter paper indicated the existence of oils and resins in plant test samples.

### Quantitative phytochemical constituent determination

The extract samples of *P.jacquemontiana* showing the presence of alkaloids, phenols, flavonoids, tannins and saponins in qualitative analysis were further quantified employing standard procedures.

#### Alkaloid quantification

Methodology of [22] was followed for the quantitative determination of alkaloids. 1 g of sample was weighed in a 250 ml beaker and 150 ml 10% acetic acid prepared in ethanol was added to it. The solution mixture was covered and allowed to stand for a time interval of 4 h. After the required time interval, the mixture was filtered and the resultant filtrate was concentrated on water-bath to reduce its volume up to a quarter of its original volume. Conc.NH_4_OH was added to the extract sample dropwise until complete precipitation. The solution was allowed to settle, precipitate collected, washed with dilute NH_4_OH and filtered. The residue obtained was completely dried and weighed to calculate the percentage of alkaloids in the test sample.

#### Phenol quantification

First of all, fat-free sample was prepared by defatting 1 g sample with 100 ml di-ethyl ether employing soxhlet apparatus for a time interval of 2 h. Phenol quantification was done by utilizing spectrophotometric method. The fat-free sample was boiled for 15 min with ether (50 ml) for complete extraction of phenolic components. 5 ml extract was pipetted into 50 ml-flask, and 10 ml distilled water was added. After this, 2 ml NH_4_OH solution was added followed by addition of 5 ml conc. amyl alcohol. After making samples up to the mark, they were left to react for 30 min to develop colour which was measured at 505 nm against standard curve of gallic acid. Results were quantified in the form of GAE (mg of Gallic Acid Equivalents) per gram of dry plant extract/fraction.

#### Flavonoid quantification

Procedure of [23] was followed for quantification of flavonoids in the test samples. For this purpose, 1 g plant sample was repeatedly extracted with 100 ml 80% aqueous methanol kept at room temperature and finally filtered through Whatman # 42 filter paper (125 mm). The filtrate was transferred into crucible and left for complete evaporation over water bath. After drying, the sample was weighed until constant weight obtained.

#### Tannin quantification

Tannin content was determined by following the methodology of [24] with slight modifications. According to this, 500 mg sample was weighed in 50 ml plastic bottle. 50 ml distilled water was added and left for shaking on mechanical shaker for a time period of 1 h. This was filtered and made up to the mark in 50 ml volumetric flask. 5 ml of filtrate was pipetted out in a test tube and mixed thoroughly with 2 ml FeCl_3_ (0.1 M) in HCl (0.1 N) and potassium ferrocyanide (0.008 M). The absorbance at 120 nm was measured spectrophotometrically against standard curve of gallic acid. Results were quantified in the form of GAE (mg of Gallic Acid Equivalents) per gram of dry plant extract/fraction [25].

#### Saponin quantification

Determination Procedure of [26] was used for saponin quantification. 1 g plant sample was dispersed in 150 ml of 20% aqueous ethanol. The suspension was heated for 4 h on water-bath at 55 °C with continuous stirring. The mixture after filtration was re-extracted with another 150 ml 20% aqueous ethanol. The combined extracts were reduced over water-bath to 40 ml by heating at 90 °C. The concentrate was transferred to separating funnel after addition and vigorous shaking with 20 ml diethyl ether. The aqueous layer obtained was collected and ether layer discarded. This purification step was repeated. 50 ml n-butanol was added and combined n-butanol extracts were washed twice with 10 ml aqueous NACl (5%). The remaining solution was transferred to water-bath and heated till complete evaporation. After evaporation, samples were dried in oven to achieve a constant weight. Saponin content was calculated as percentage yield of the sample.

### Antibacterial screening

For antibacterial screening total of 19 clinical bacterial strains were tested in this experiment. Bacterial strains comprised of 7 multidrug resistant (MDR) bacterial strains viz. *Staphylococcus aureus* (3884), *Staphylococcus aureus* (6301), *Staphylococcus aureus (9861), Staphylococcus lugdunensis* (6197), *Klebsiella pneumoniae* (87005) ESBL, *Klebsiella pneumoniae* (82431), and *Escherichia coli* (52321). There were 12 other clinical isolates including; *Staphylococcus aureus* MRSA (12861), *Staphylococcus aureus* (31414), *Staphylococcus aureus* (5764), *Staphylococcus lugdunensis* (4338), *Pseudomonas aeruginosa* (27853), *Coagulase negative staphylococci* (12731), *Escherichia coli* (22244), *Klebsiella specie* (34529), and *Klebsiella pneumoniae* (35967) whereas 3 bacterial strains isolated from the environmental setup; *Escherichia coli “a”*, *Escherichia coli “b”*, and *Klebsiella pneumoniae “a”*, tested in this experiment. The bacterial strains were acquired from the Department of Microbiology, Quaid-i-Azam University Islamabad.

For the determination of antibacterial activity all the plant samples were dissolved in DMSO to make a final concentration of 50 mg/ml. Antibacterial activities were carried out by disc diffusion method [27]. All the bacterial strains were cultured overnight in nutrient agar medium (NA) at 37 °C prior to experimentation. The following day, about 100 μl of saline suspension containing 10^8^ CFU/ml of bacteria were spread on nutrient agar medium plates. The particular test strain inoculum was uniformly distributed on the petri plates by swabbing with sterile cotton swabs in three dimensions to ensure the growth of that particular strain on the plate. Sterile filter paper discs (6 mm in diameter) were impregnated with 20 μl of each test sample (1000 μg/disc), dried and placed over the surface of media. Negative controls were prepared by using the same solvent in which plant extract samples were prepared. The bacterial inoculated plates were incubated at 37 °C for 24 h. The assays were performed in triplicates against each bacterial strain. After the specified time, diameters of zones of inhibition formed around discs were measured in millimeters (mm) to determine the activity of test samples against different strains [28]. 8-13 mm: low inhibition; 14-19 mm: moderate inhibition; ≥ 20 mm: high inhibition [29].

#### Bacterial MIC and MBC determination

The MIC (minimal inhibitory concentration) and MBC (minimal bactericidal concentration) was assessed for the effective plant samples tested during the disc diffusion assay. Bacterial tests were performed in nutrient agar broth (NAB) and about 12 h-old bacterial cultures were used and their suspension turbidity was compared with the turbidity of 0.5 McFarland standards. The plant samples were first thoroughly dissolved in 2.5% DMSO and then diluted to make a concentration 512 μg/ml as the highest concentration tested. Further two-fold serial dilutions were prepared to achieve a concentration ranging from 8 to 512 μg/ml in sterile test tubes. MIC values of the plant samples were calculated against all bacterial strains isolates by a combination of micro-well dilution assay and *p*-Iodonitrotetrazolium chloride 0.2% (INT) calorimetric assay [30, 31].

Briefly, 96-well plates were prepared by distributing 95 μl of nutrient broth into each well followed by 5 μl of inoculum. From the primarily formed stock solutions of plant extracts having a concentration 512 μg/ml, a 100 μl was picked and dispensed into the first wells. Further six consecutive wells downward were dispensed with 100 μl each from their serial dilutions and the last well having no compound except adequate amount of nutrient broth, DMSO and 5 μl of inoculum, which were used as negative control. The final volume of each well was made up to 200 μl only. Cefixime was prepared in nutrient broth at the concentration ranging from 2 to 128 μg/ml and used as positive control standard drug. Sterile plate sealer was used to fully cover the plate and then a plate shaker at the speed of 300 rpm was used to mix the contents of each well for about 20 s. The bacterial inoculated plates were incubated for 24 h at 37 °C and 40 μl of INT (0.2 mg/ml) was added to it the next day followed by 30 min incubation period. Viable bacteria present in the samples turned the yellow dye into a pink colour. MIC was demarcated as the sample concentration which prohibited the medium to change colour thus demonstrating inhibition of bacterial growth.

For MBC determination broth was picked from each well and spread on nutrient agar medium for a time interval of 24 h at 37 °C. The MBC was defined as the least possible concentration of the plant extract samples to completely kill the inoculated microorganisms [32]. Negative control included NAB with DMSO whereas cefixime served as positive control. Furthermore, MBC/MIC ratio was calculated against each bacterial strain depicting whether the antibacterial effect is bactericidal or bacteriostatic [33].

$$ 1\le \left(\mathrm{MBC}/\mathrm{MIC}\right)\le 2=\mathrm{Bactericidal}\ \mathrm{effect} $$

$$ 4\le \left(\mathrm{MBC}/\mathrm{MIC}\right)\le 16=\mathrm{Bacteriostatic}\ \mathrm{effect} $$

### Antifungal screening

The antifungal and anti-yeast activity of the plant samples was estimated by disc diffusion assay [27]. Prior to experimentation, the fungal and yeast strain was cultured overnight in Sabouraud dextrose agar (SDA) medium at 30 °C. The following day, 100 μl of distilled water suspension containing 10^4^ spore/ml of fungus and 10^6^ CFU/ml of yeast were spread on Sabouraud dextrose agar medium plates. Fungal isolates included 7 fungal strains viz. *Aspergillus niger* (ATCC 6275), *Mucor piriformis* (ATCC 52554)*, Fusarium solani* (ATCC 36031), *Aspergillus flavus* (ATCC 204304), *Wickerhamomyces anomalus* (KU949595), *Wickerhamomyces anomalus* (KU949596), *Deboromyces hansenii* and a yeast *Candida albicans* (90028) obtained from the American Type Culture Collection (ATCC) or from the environmental setup. The fungal strains were acquired from the Department of Microbiology, Quaid-i-Azam University Islamabad. Briefly, sterile discs 6 mm diameter containing 1000 μg/disc of each extract sample was placed over the surface of media. DMSO was used as the negative control. The fungal and yeast inoculated plates were incubated at 28 °C for 72 h and 48 h, respectively. The antifungal and anti-yeast activity was assessed in terms of inhibition zones (mm) after a specified period [28]. The experiment was performed in triplicate. 8-13 mm: low inhibition; 14-19 mm: moderate inhibition; ≥ 20 mm: high inhibition [29].

#### Fungal MIC and MFC determination

The MIC (minimal inhibitory concentration) and MFC (minimal fungicidal concentration) were determined for the effective test samples. The media used for MIC and MFC determination was Sabouraud dextrose broth (SDB). Fungal cultures and concentration of the test samples used were similar as above for bacterial studies [30, 31]. For determination of fungal MIC, the inoculated plates were incubated for 48 h at 25-28 °C. Absorbance was taken at 600 nm by subjecting the plate to a universal microplate reader and microbial growth was determined. The MIC was defined to be the lowest possible concentration of the plant extract samples under study to clearly inhibit visible growth of microorganisms. To determine the MFC, broth was picked from each well and spread/plated on Sabouraud dextrose agar medium for 48 h at 25-28 °C. The MFC was defined as the least possible concentration of the plant extract samples to completely kill the inoculated microorganisms [32]. These assays were performed in triplicates. Negative control included SDB medium with DMSO while clotrimazole served as positive control standard. Furthermore, MFC/MIC ratio was calculated against each fungal and yeast strain depicting whether the antifungal effect is fungicidal or fungistatic [33].

$$ 1\le \left(\mathrm{MFC}/\mathrm{MIC}\right)\le 2=\mathrm{Fungicidal}\ \mathrm{effect} $$

$$ 4\le \left(\mathrm{MFC}/\mathrm{MIC}\right)\le 16=\mathrm{Fungistatic}\ \mathrm{effect} $$

### Statistical analysis

The data was expressed as mean ± standard deviation. All the experimental assays were performed in triplicate.

## Results

### Plant yield and fractionation

The extraction yield of methanol extract of *P. jaqcuemontiana* leaves (PJM) was approximately 95 g. Upon fractionation (50 g) five different fractions (PJH, PJC, PJE, PJB, and PJA) were formed and their percentage extraction yield was 30, 2, 36, 12 and 18% as presented in Table 1, respectively .

**Table 1:** Extraction yield of *P. jacquemontiana* methanol extract and its corresponding fractions

Plant sample	Percentage yield (%)
PJM	50^a^
PJH	30
PJC	2
PJE	36
PJB	12
PJA	18

PJM (*P. jacquemontiana* methanol fraction), PJH (*P. jacquemontiana* n-hexane fraction), PJC (*P. jacquemontiana* chloroform fraction), PJE (*P. jacquemontiana* ethyl acetate fraction), PJB (*P. jacquemontiana* butanol fraction), PJA (*P. jacquemontiana* aqueous fraction)

^a^Yield of PJM in grams based on dry powder weight; fraction yield dependent upon PJM yield

### Phytochemical qualitative study

The phytochemical analysis study of PJM and its derived fractions revealed important medicinal constituents present within the sample as illustrated in Table 2. Qualitative analysis confirmed the existence of coumarins, flavonoids, tannins, alkaloids, sterols, betacyanins, triterpenoids, phlobatannins, steroids, phenols, glycosides, saponins, quinones, acid, vitamin C, proteins, oils and resins in PJM. Presence of anthraquinones, phytosteroids, carbohydrates and anthocyanin were not established in PJM. Alkaloids, triterpenoids, quinones, acid, vitamin C, proteins, oils and resins were the classes present in PJH. PJC comprised of nearly all chemical classes studied except sterols, steroids, phlobatannins, phytosterols, saponins, phytosteroids, carbohydrates, anthraquinone and anthocyanin. Coumarins, alkaloids, saponins, tannins, phlobatannins, steroids, phytosterols, glycosides, quinones, acid, vitamin C, proteins, carbohydrates, sterols, oils and resins marked their existence in PJE. Terpenoids, triterpenoids, anthraquinones, phenols, phytosteroids, sterols and anthocyanin were absent in PJB whereas all other remaining chemical classes were present. PJA marked the absence of terpenoids, triterpenoids, anthraquinones, phlobatannins, phenols, alkaloids, carbohydrates, phytosteroids, saponins and anthocyanin and presence of coumarins, flavonoids, tannins, steroids, sterols, phytosterols, glycosides, quinones, acid, vitamin C, proteins, betacyanin, oils and resins as depicted in Table 2, respectively.

**Table 2:** Qualitative phytochemical analysis of *P. jacquemontiana* leaves methanol extract and its derived fractions

Phytochemical	PJM	PJH	PJC	PJE	PJB	PJA
Terpenoids	+	+	+	–	–	–
Triterpenoids	+	+	+	–	–	–
Coumarins	+	–	+	+	+	+
Flavonoids						
Alkaline reagent test	+	–	+	–	+	+
FeCl_3_ test	+	–	+	–	+	+
Tannins						
Alkaline reagent test	+	–	+	+	+	+
FeCl_3_ test	+	–	+	+	+	+
Condensed Tannins	+	–	–	+	+	+
Hydrolysable tannins	–	+	+	–	–	–
Phlobatannins	+	–	–	+	+	–
Steroids	+	–	–	+	+	+
Phytosteroids	–	–	–	+	+	+
Anthraquinones	–	–	–	–	–	–
Phenols	+	–	+	–	–	–
Alkaloids						
Mayer’s test	+	+	+	+	+	–
Hager’s test	+	+	+	+	+	–
Glycosides	+	+	+	+	+	+
Saponins	+	–	–	+	+	–
Sterols	+	–	–	+	–	+
Quinones	+	+	+	+	+	+
Acids	+	+	+	+	+	+
Vitamin C	+	+	+	+	+	+
Proteins						
Xanthoproteic test	+	+	+	+	+	+
Biuret test	+	+	+	+	+	+
Carbohydrates	–	–	–	+	+	–
Oils and Resins	+	+	+	+	+	+
Anthocyanin	–	–	–	–	–	–
Betacyanin	+	–	+	–	+	+

*PJM* methanol extract of *P. jacquemontiana* leaves, *PJH* n-hexane fraction of PJM, *PJC* chloroform fraction of PJM; *PJE* ethyl acetate fraction of PJM, *PJB* n-butanol fraction of PJM, *PJA* residual aqueous fraction of PJM. (+) constituent present, (−) constituent absent

### Phytochemical quantitative study

Based on preliminary phytochemical qualitative test results obtained, the quantitative estimation of some major phytochemicals including alkaloids, flavonoids, phenols, saponins and tannins were carried out in those plant extract samples where their presence was marked positive. Comparative quantitative estimation of different PJM derived fractions is shown in Table 3. Highest alkaloid percentage yield/g of sample was presented by PJE (22.5 ± 0.5) consecutively followed by PJM (12.5 ± 0.17). PJC and PJB displayed alkaloid percentage values of 7.7 ± 0.14 and 5.7 ± 0.36, respectively whereas least value was shown by PJH (0.71 ± 0.21) having minimal alkaloid percentage yield/g of sample. Flavonoid percentage yield/g of sample was marked highest in PJM (7.4 ± 0.14) followed by PJB (6.6 ± 0.42), PJA (4.6 ± 0.11) and PJC (3.5 ± 0.07), respectively. Saponin content expressed as percentage yield/g of sample exhibited maximum values for PJE (5.5 ± 0.28) followed closely by PJM (4.4 ± 0.42) whereas minimal values was shown by PJB (1.8 ± 0.11), respectively. Apart from this, phenols were depicted maximum in PJM showing a value of 611.5 ± 2.18 mg GAE/ g of extract compared to PJC showing a value of 342.8 ± 1.69 mg GAE/ g of extract whereas tannins were minimum in PJA (110.5 ± 0.74 mg GAE/ g of extract) followed by PJM (159.4 ± 1.85 mg GAE/ g of extract) and maximum in PJE (244.3 ± 2.03 mg GAE/ g of extract), closely followed by PJB (231.6 ± 1.93 mg GAE/ g of extract) and PJC (203.8 ± 2.67 mg GAE/ g of extract), respectively.

**Table 3:** Quantitative phytochemical analysis of *P. jacquemontiana* leaves methanol extract and its derived fractions

Percentage (%) yield per gram				mg of GAE/ g of extract	
Plant extracts	Alkaloids	Flavonoids	Saponins	Phenols	Tannins
PJM	12.5 ± 0.17	7.4 ± 0.14	4.4 ± 0.42	611.5 ± 2.18	159.4 ± 1.85
PJH	0.71 ± 0.21	NT	NT	NT	NT
PJC	7.7 ± 0.14	3.5 ± 0.07	NT	342.8 ± 1.69	203.8 ± 2.67
PJE	22.5 ± 0.5	NT	5.5 ± 0.28	NT	244.3 ± 2.03
PJB	5.7 ± 0.36	6.6 ± 0.42	1.8 ± 0.11	NT	231.6 ± 1.93
PJA	NT	4.6 ± 0.11	NT	NT	110.5 ± 0.74

Mean ± SD (*n* = 3), *PJM P. jacquemontiana* methanol fraction, *PJH P. jacquemontiana* n-hexane fraction, *PJC P. jacquemontiana* chloroform fraction, *PJE P. jacquemontiana* ethyl acetate fraction, *PJB P. jacquemontiana* butanol fraction, *PJA P. jacquemontiana* aqueous fraction, *NT* Not Tested

### Antibacterial activity of *P. jacquemontiana*

In vitro antibacterial activity of *P. jacquemontiana* was evaluated qualitatively and quantitatively against the selected microorganisms by the manifestation or absence of zones of inhibition, inhibition diameters, MIC and MBC values. According to the initial bacterial screening results specified in Table 4, the methanol fraction of *P. jacquemontiana* (PJM) showed strong activity against all the bacterial strains under study. The obtained results showed the variability of antibacterial potential of PJM fraction towards different gram positive and gram negative selected microorganisms. The most sensitive bacterial strain proved to be *Staphylococcus lugdenesis*, a gram positive MDR (6197) followed by a gram negative MDR, *ESBL-Klebsiella pneumoniae* (87005) displaying zones of inhibition 35.5 ± 1.32 mm and 35.33 ± 0.57 mm, respectively. Least activity was shown against strain *Klebsiella pneumoniae* (35967) exhibiting a zone diameter of 22.16 ± 0.76 mm. PJH and PJC showed activity against nearly all tested bacterial strains excluding *Escherichia coli “a”, Klebsiella pneumoniae* and *Escherichia coli “b”* (all isolated from environmental setup). PJC also failed to show activity against *Staphylococcus lugdenesis* (4338). The highest and lowest zone inhibition diameter presented by PJH was 34 ± 1.0 mm against MDR *ESBL-Klebsiella pneumoniae* (87005) and 13.16 ± 0.28 mm against strain *Escherichia coli* (22244), respectively. The most sensitive microorganism tested against PJC was *Staphylococcus aureus* (31414), exhibiting a zone of inhibition 30.33 ± 1.44 mm and the least sensitive strain was *Escherichia coli* (22244) and *Pseudomonas aeruginosa* (27853) both having similar zone of inhibition; 14 ± 1.0 mm and 14 ± 2.0 mm, respectively. Ethyl acetate fraction of *P. jacquemontiana* (PJE) exhibited strong antibacterial activity against all tested microorganisms displaying maximum zone of inhibition 28 ± 1.32 mm against a gram positive MDR *Staphylococcus aureus* (6301) and minimum zone of inhibition 19.66 ± 1.52 mm against *Pseudomonas aeruginosa* (27853). Butanol fraction of the plant (PJB) inhibited all bacterial test organisms showing maximum potency 33.33 ± 0.57 mm against gram negative MDR *Klebsiella pneumoniae* (82431) and least potency 18.33 ± 0.57 mm against *Pseudomonas aeruginosa* (27853). Aqueous fraction of the plant (PJA) was tested against all selected microorganisms but it did not show satisfactory zones of inhibition.

**Table 4:** Antibacterial screening of *P. jacquemontiana* extract against gram positive and gram negative bacteria

	Extract/Fractions (1000 μg/disc)					
Isolate (s)	PJM	PJH	PJC	PJE	PJB	PJA
Staphylococcus aureus						
MDR (6301)	24.16 ± 1.25	27.66 ± 0.28	25.16 ± 1.04	28 ± 1.32	24.16 ± 1.04	–
MDR (3884)	31.16 ± 2.02	29 ± 1.0	24.83 ± 0.28	26.5 ± 0.5	28.66 ± 0.28	–
MRSA (12861)	22.5 ± 1.32	23.16 ± 1.25	25 ± 0.5	26 ± 1.0	25.33 ± 1.04	–
MDR (9861)	25.16 ± 0.04	22.5 ± 0.5	26.33 ± 1.25	26.5 ± 0.86	28.83 ± 0.76	–
(5764)	29 ± 1.0	26 ± 1.0	27.33 ± 0.57	27 ± 1,0	23.5 ± 0.5	–
(31414)	26 ± 1.0	32 ± 0.86	30.33 ± 1.44	22.5 ± 0.5	25 ± 1.0	–
Staphylococcus lugdenesis						
MDR (6197)	35.5 ± 1.32	28.5 ± 0.5	24.83 ± 1.04	26.5 ± 0.5	26.83 ± 0.28	–
(4338)	24 ± 1.0	18 ± 1.0	-	25.66 ± .57	20.5 ± 0.86	–
Escherichia coli						
MDR (52331)	27.83 ± 0.76	28.83 ± 0.28	24.83 ± 1.25	24.33 ± 0.57	31.16 ± 0.76	–
(22244)	28 ± 0.5	13.16 ± 0.28	14 ± 1.0	27.83 ± 0.76	23.5 ± 0.86	–
*“a”*	23.5 ± 0.5	-	-	24.16 ± 0.76	18.33 ± 1.04	–
*“b”*	24.5 ± 0.5	-	-	25.66 ± 0.28	19 ± 1.0	–
Klebsiella pneumoniae						
MDR (82431)	30 ± 1.0	26 ± 2.0	26.66 ± 0.28	25.16 ± 0.28	33.33 ± 0.57	–
*ESBL* MDR (87005)	35.33 ± 0.57	34 ± 1.0	29.5 ± 0.5	26 ± 1.0	28.16 ± 0.76	–
(34529)	27.83 ± 0.28	17.16 ± 1.04	18 ± 1.0	21.83 ± 1.04	20 ± 1.0	–
(35967)	22.16 ± 0.76	-	-	20.83 ± 1.04	19 ± 0.86	–
*“a”*	26 ± 2.0	-	-	23.83 ± 0.57	19 ± 0.5	–
Pseudomonas aeruginosa						
(27853)	25.16 ± 0.76	21.5 ± 0.5	14 ± 2.0	19.66 ± 1.52	18.33 ± 0.57	–
Coagulase negative staphylococci						
(12731)	28 ± 2.0	27.33 ± 0.57	22 ± 1.0	25.16 ± 1.04	26.16 ± 0.76	–

Zone of inhibition (mm) are expressed as mean ± SD (n = 3). 8-13 mm: low inhibition; 14-19 mm: moderate inhibition; ≥ 20 mm: high inhibition. -: no zone of inhibition; *NT* not tested, *PJM* methanol extract of *P. jacquemontiana* leaves, *PJH*: n-hexane fraction of PJM, *PJC* chloroform fraction of PJM, *PJE* ethyl acetate fraction of PJM, *PJB* n-butanol fraction of PJM, *PJA* residual aqueous fraction of PJM

#### MIC and MBC of *P. jacquemontiana*

The MIC and MBC values of PJM against 19 bacterial strains examined showed to range between 8 and 64 μg/ml and 32-256 μg/ml, respectively (Tables 5 and 6). PJH showed an MIC and MBC value ranging between 8 and 128 μg/ml and 64-256 μg/ml. PJC indicated an MIC value range of 32-512 μg/ml and an MBC value in the range of 128-512 μg/ml, respectively. PJE almost similar to PJM specified its MIC value ranging 8-64 μg/ml and MBC ranging 32-128 μg/ml. PJB designated its MIC and MBC value falling in the range of 8-512 μg/ml and 64-512 μg/ml, respectively. Cefixime exhibited an MIC value in the range of 2-128 μg/ml and an MBC value ranging between 8 and 128 μg/ml. MBC/MIC was also calculated for each extract tested against the bacterial strains.

**Table 5:** MIC and MBC of *P. jacquemontiana* extracts against gram positive and gram negative bacteria

Extract/Fractions (μg /ml)									
	PJM			PJH			PJC		
Isolate(s)	MIC	MBC	MBC/MIC	MIC	MBC	MBC/MIC	MIC	MBC	MBC/MIC
Staphylococcus aureus									
MDR (6301)	32	64	02	64	256	04	128	512	04
MDR (3884)	16	64	04	128	256	02	128	256	02
MRSA (12861)	08	32	04	16	128	08	32	128	04
MDR (9861)	32	64	02	32	128	04	128	256	02
(5764)	16	128	08	128	256	02	64	256	04
(31414)	08	32	04	16	128	08	32	256	08
Staphylococcus lugdenesis									
MDR (6197)	32	128	04	64	128	02	512	512	01
(4338)	16	64	04	64	256	04	–	–	–
Escherichia coli									
MDR (52331)	32	128	04	64	128	02	256	512	02
(22244)	08	64	08	32	64	02	64	256	04
*“a”*	08	32	04	–	–	–	–	–	–
*“b”*	08	32	04	–	–	–	–	–	–
Klebsiella pneumoniae									
MDR (82431)	64	256	04	128	512	04	128	512	04
*ESBL* MDR (87005)	16	64	04	64	256	04	256	512	02
(34529)	08	32	04	08	64	08	32	128	04
(35967)	08	32	04	–	–	–	–	–	–
*“a”*	08	64	08	–	–	–	–	–	–
Pseudomonas aeruginosa									
(27853)	08	64	08	32	128	04	128	256	02
Coagulase negative staphylococci									
(12731)	64	128	02	64	256	04	128	512	04

*MIC* minimum inhibitory concentration, *MBC* minimum bactericidal concentration, *PJM* methanol extract of *P. jacquemontiana* leaves, *PJH* n-hexane fraction of PJM, *PJC* chloroform fraction of PJM; −: no zone of inhibition

**Table 6:** MIC and MBC of *P. jacquemontiana* extracts and cefixime against gram positive and gram negative bacteria

	Extract/Fractions (μg/ml)						Antibiotic used (μg/ml)		
	PJE			PJB			Cefixime		
Clinical Isolate(s)	MIC	MBC	MBC/MIC	MIC	MBC	MBC/MIC	MIC	MBC	MBC/MIC
Staphylococcus aureus									
MDR (6301)	32	64	02	128	256	02	16	64	04
MDR (3884)	08	32	04	64	256	04	08	16	02
MRSA (12861)	08	64	08	32	128	04	64	64	01
MDR (9861)	32	128	04	64	128	02	64	128	02
(5764)	32	128	04	64	256	04	32	64	02
(31414)	16	64	04	08	64	08	08	16	02
Staphylococcus lugdenesis									
MDR (6197)	32	128	04	512	512	01	32	32	01
(4338)	16	64	04	32	128	04	08	32	04
Escherichia coli									
MDR (52331)	64	128	02	128	128	01	128	128	01
(22244)	08	32	04	16	64	04	16	32	02
*“a”*	08	64	08	32	128	04	16	32	02
*“b”*	08	64	08	32	128	04	08	32	04
Klebsiella pneumoniae									
MDR (82431)	32	256	08	32	128	04	128	128	01
*ESBL* MDR (87005)	16	64	04	32	256	08	64	128	02
(34529)	08	32	04	08	64	08	08	16	02
(35967)	08	64	08	16	128	08	64	128	02
*“a”*	08	64	08	16	64	04	02	08	04
Pseudomonas aeruginosa									
(27853)	16	64	04	08	128	16	08	64	08
Coagulase negative staphylococci									
(12731)	32	128	04	32	128	04	64	128	02

*MIC* minimum inhibitory concentration, *MBC* minimum bactericidal concentration, *PJE* ethyl acetate fraction of PJM, *PJB* n-butanol fraction of PJM, *PJA* residual aqueous fraction of PJM

### Antifungal activity of *P. jacquemontiana*

Antifungal activity of all the fractions of *P. jacquemontiana* evaluated by in vitro disc diffusion method against 7 fungal and 1 yeast strain indicated in Table 7. Zones of inhibition were formed and their diameters were measured in mm against selected microorganisms in 3 fractions of the plant. PJM, PJE and PJB showed significant zone of inhibition whereas PJH, PJC and PJA did not show any activity. The most susceptible microorganism towards PJM was *Aspergillus flavus* (ATCC 204304) giving a zone of inhibition 29 ± 1.0 mm while the least susceptible was *Fusarium solani* (ATCC 36031) with inhibition zone of 20 ± 1.25 mm. PJE inhibited the growth of *Wickerhamomyces anomalus* (KU949595) at its highest by forming a diametric zone of inhibition 28 ± 1.0 mm while the minimum zone 18.66 ± 0.28 mm was formed against *Mucor piriformis* (ATCC 52554). In case of PJB, the most sensitive strain tested against it was *Candida albicans*, 25.83 ± 0.28 mm while the least sensitive proved to be *Aspergillus flavus* (ATCC 204304) forming a diameter of 15.16 ± 1.04 mm.

**Table 7:** Antifungal screening of *P. jacquemontiana* extract/fractions

	Extract/fractions (1000 μg/disc)					
Fungal isolates	PJM	PJH	PJC	PJE	PJB	PJA
*F. solani* ATCC 36031	20.33 ± 1.25	–	–	24.83 ± 1.04	17.33 ± 1.52	–
*A. niger* ATCC 6275	22.66 ± 2.08	–	–	21.33 ± 1.52	23.66 ± .57	–
*A. flavus* ATCC 204304	29 ± 1.0	–	–	23.33 ± 1.25	15.16 ± 1.04	–
*M. piriformis* ATCC 52554	26.66 ± 0.57	–	–	18.66 ± 0.28	24 ± 2.0	–
*C. albicans* (90028)	23.33 ± 1.25	–	–	25.5 ± 0.5	25.83 ± 0.28	–
*D. hansenii*	25 ± 1.0	–	–	26.83 ± 0.76	21.66 ± 0.28	–
*W. anomalus* (KU949596)	27.33 ± 0.76	–	–	25.16 ± 1.60	22.5 ± 1.32	–
*W. anomalus* (KU949595)	25.33 ± 1.52	–	–	28 ± 1.0	19.83 ± 1.25	–

Zone of inhibition (mm) are expressed as mean ± SD (n = 3). 8-13 mm: low inhibition; 14-19 mm: moderate inhibition; ≥20 mm: high inhibition. -: no zone of inhibition. *PJM* methanol extract of *P. jacquemontiana* leaves, *PJH* n-hexane fraction of PJM, *PJC* chloroform fraction of PJM, *PJE* ethyl acetate fraction of PJM, *PJB* n-butanol fraction of PJM, *PJA* residual aqueous fraction of PJM

#### MIC and MFC of *P. jacquemontiana*

The MIC and MFC values of three fractions (PJM, PJE and PJB) of *P. jacquemontiana* and clotrimazole were evaluated as shown in Table 8. PJM presented strongest antifungal activity against the test strains hence displaying more minimal MIC and MFC values compared to the other fractions. It’s MIC and MFC values ranged within 8-64 μg/ml and 32-256 μg/ml. PJE fraction showed almost similar results displaying an MIC in the range of 8-64 μg/ml whereas MFC ranging from 32 to 512 μg/ml was presented. PJB exhibited an MIC and MFC in the range of 32-128 μg/ml and 64-512 μg/ml, respectively. MIC/MFC values were also calculated for each plant extract tested against the fungal species.

**Table 8:** Antifungal screening of *P. jacquemontiana* extracts and clotrimazole

	Extract/Fractions (μg/ml)									Standard (μg/ml)		
	PJM			PJE			PJB			Clotrimazole		
Clinical Isolate(s)	MIC	MFC	MFC/MIC	MIC	MFC	MFC/MIC	MIC	MFC	MFC/MIC	MIC	MFC	MFC/MIC
*F. solani* ATCC 36031	32	128	04	32	64	02	32	128	04	04	08	02
*A. niger* ATCC 6275	16	32	02	16	128	08	128	256	02	32	64	02
*A. flavus* ATCC 204304	08	32	04	32	256	08	128	512	04	32	128	04
*M. piriformis* ATCC 52554	32	128	04	08	128	16	32	64	02	08	16	02
*C. albicans* (90028)	64	256	04	64	256	04	64	128	02	16	64	04
*D. hansenii*	64	256	04	64	512	08	32	512	16	64	128	02
*W. anomalus* (KU949596)	08	64	08	32	64	02	32	256	08	08	32	04
*W. anomalus* (KU949595)	16	32	02	08	32	04	64	512	08	16	64	04

*MIC* minimum inhibitory concentration, *MFC* minimum fungicidal concentration, *PJM* methanol extract of *P. jacquemontiana* leaves, *PJE* ethyl acetate fraction of PJM, *PJB* n-butanol fraction of PJM, *PJA* residual aqueous fraction of PJM

## Discussion

Plants can contribute to the advancement of novel chemo-preventive agents as they have been proven essential in forming potentially useful structures. The initial step to this achievement is performing antibacterial activities [34]. Plants are the gifts of nature for producing a wide collection of phytochemicals that are related to stress or defense mechanisms as well as antimicrobial activities and play significant role [35]. This diverse existence of rich phytochemicals has become popular due to its enhanced defense mechanisms alongside a variety of microorganisms, nematodes, insects and other plants [36]. The existing literature suggests that phytochemicals documented for their antimicrobial potential belong to the major subclasses phenols, flavones, quinones, flavonols, terpenoids, coumarins, tannins, essential oils, polyamines, glycosides, alkaloids and many more [37, 38]. Although many classes of phytochemicals have been reported having antimicrobial abilities yet they have not been recognizes as therapeutic agents by the medical communal [39]. Secondly, to successfully predict which botanical compounds can be present in the plant material, it is important to keep in view the type of solvent used for extraction procedure. Traditional practitioners made the use of water as a solvent but studies reported proved that methanol extracts were undoubtedly much better and hence more powerful. This is recognized due to the better solubility of the plant active metabolites in organic solvents [40]. These clarifications can be rationalized by the escalating polarity of the compounds extracted by different solvents and their intrinsic bioactivity.

Our present study revealed good antibacterial and antifungal effect of plant extracts towards a panel of microorganisms under study. This may be due to the rich diversity of phytochemicals such as flavonoids, saponins, alkaloids, phenols and tannins present in the plant extracts in higher amounts as well as the type of solvent used to completely extract these bioactive compounds present within the plant. Results of quantitative analysis indicate highest phenol content in PJM, which comply to the statement that methanol, is undoubtedly considered the best solvent for extraction of phenolic compounds due to better solubility and polarity of the solvent [41]. Similar to the study conducted by [41], in which the methanol extract of *thymus vulgaris* showed maximum phenolic compounds per mg of GAE/g extract, our study also displayed similar results in case of PJM extract in comparison to PJC extract. A wide range of phenolic compounds are well-known for their antimicrobial potential, which may describe the potent antibacterial and antifungal activities of PJM in the present study. Apart from this, flavonoids are a chemical class most often correlated with antimicrobial efficacy of herbal extracts. Many flavonoids are well reported showing anti-infective effects by forming complexes with different extracellular proteins and proteins residing in bacterial cell wall [42]. Our study showed PJM to contain the highest flavonoid contents in addition to highest phenolic contents, which is in accordance with the findings of [42] who reported the methanol extract to have maximum phenolic and flavonoid levels, and displaying more pronounced antibacterial potential compared to other solvent extracted samples. Antibacterial activity was found to be more pronounced against gram positive bacteria compared to gram negative. This confides itself to the previous reports showing that plant extracts have better potential against gram positive microorganisms than gram negative [43, 44]. Gram positive and negative bacteria have different cell envelope structures. The outer membrane surrounding cell wall is found in negative bacteria only which will act as an impermeable membrane for the passage of substances through it whereas no outer membrane lining cell wall is present in gram positive bacterium. This allows the substances to easily access it as it is permeable to most substances. Generally, the plant extracts capable of inhibiting gram negative bacteria equally like the gram positive bacteria can be accredited to its total phenolic content, which when higher can act as broad-spectrum antibacterial agent whereas when lower then shows almost no antibacterial activity [45]. On a similar notice, one of the known resistant gram negative bacterial strain called *Pseudomonas aeruginosa* (27853), showed maximum sensitivity towards PJM compared to other extracts tested, which may be possible due to high phenol content present in PJM. Moreover it can be said that foods rich in polyphenols may significantly reduce the risk of various health problems due to their anti-mutagenic, ant-inflammatory, antioxidant, and antibacterial properties [46]. Another diverse studied plant phytochemical is saponins, which have also been claimed to possess antibacterial activity. Our study revealed saponin content to be present in PJM, PJE and PJB fractions only. All these plant extract samples showed promising antibacterial activity compared to other extracts (PJH, PJC and PJA) in which their presence was not detected. It can be said that high content of saponins and tannins present in plant extracts could be considered as the basis for its antimicrobial property as claimed previously that saponin and tannin rich plants have profound antimicrobial activity [47]. Tannins are one of the important chemical groups of compounds having constitutive structural function present in almost every plant part including leaves, fruits, roots, bark and wood [48]. They account for the astringent taste of unripe fruits or wine and are constituents of the different colors seen in flowers and autumn weather leaves [49]. Tannins have been reported to disrupt the cytoplasmic membrane, interrupt the PMF (proton motive force), active transport, coagulation of cell substances and electron flow [50]. This can be a reason that almost all the plant extract fractions of *P. jacquemontiana* having high tannin content were able to inhibit all the tested organisms especially the gram positive microorganisms, having lipopolysaccharide-deficient outer membrane and hence proved to be more susceptible towards them. Tannins can exist in two forms, either as condensed tannins or as hydrolysable tannins. Higher levels of tannins especially condensed tannins and polyphenols when present in plant extracts might be responsible for antibacterial mode of action and anticandidal activity [51]. One of the mechanisms of tannins accounting for antimicrobial potential involves extracellular enzyme inhibition. Condensed tannins have the ability to bind to cell coat polymers in strains, and form complexes resulting in inhibition of cell associated proteolytic activity of these strains. Morphology is also altered in the presence of condensed tannins implicating the role of tannin toxicity in targeting the cell wall. Moreover despite forming outer complexes with cell-coat polymers of bacterial strains, condensed tannins have the ability to penetrate through the cell wall in sufficient concentrations to react with other ultrastructural components resulting in inhibition of cell wall synthesis [52]. Condensed tannins were strongly present in PJM, PJE and PJB extracts marking their role as effective antimicrobial agents, showing broad-spectrum antimicrobial activity compared to PJH, PJC and PJA. Our results partially agree with [53], who claimed methanol extract to contain more condensed tannins compared to other extracts while our present study conducted revealed the manifestation of condensed tannins in PJM with equal intensity as in PJE and PJB, respectively. Another phytochemical class known as phlobatannins along with other chemical classes has been widely reported in the implication of causing inhibitory action against a wide range of micro-organisms when present in plants [54]. Phlobatannins were detected in PJM, PJE and PJB fractions which may account for outstanding antimicrobial activity compared to other extracts of *P.jacquemontiana*. Apart from this, another largest group of chemical compounds formed by the plants are alkaloids and they have proved their usefulness to humans as powerful pain killers hence relieving body pain [55]. Alkaloids are commonly known for their antimicrobial properties [56]. The present study indicated their presence in all plant extracts (except aqueous) paving the way for more effective research. Coumarins were also abundantly present in almost all fractions which are responsible for the stimulation of macrophages, hence playing an indirect negative role on infections [57]. It is known to inhibit *C. albicans* in vitro and is presumed to have antifungal potential also [58]. However terpenoids were absent in all fractions of this plant which are known for their effectiveness against bacteria, fungi and viruses [59–61]. Moreover the antibacterial potential of a crude extract has been demarcated as significant when its MIC value is less than 100 μg/ml, moderate when MIC< 625 μg/ml and low when MIC > 625 μg/ml [62, 63].

Fungi spoil the food materials and worsen their nutritive value. Several studies have been carried out which attribute the phenolic composition to combat bacterial pathogens [64, 65], whereas saponin detection to combat fungal pathogens [66, 67]. This can explain our plant fraction analysis in which PJM, PJE, and PJB having saponin content are showing promising antifungal results compared to the other fractions (PJH, PJC and PJA) in which saponin functional chemical group was not detected and hence it did not show activity against any fungal strain. A classification based on fungal MIC values of plant extracts was published which accredited an MIC value up to 500 μg/ml as a strong potential inhibitor, 600-1500 μg/ml was considered as moderate potential inhibitor and a value exceeding 1600 μg/ml was considered as weak potential inhibitor [68]. Triplicate results of antifungal activity showed that three fractions of *P. jacquemontiana* including PJM, PJE and PJB showed good antifungal potential displaying MIC values ≤500 μg/ml, proving them as strong potential inhibitors of fungi. It is also to be mentioned that PJA (aqueous plant extract) did not show noteworthy antibacterial and antifungal activities compared to the other extracts. This is in compliance with the findings of [69], who concluded that methanol extract of *Juniperus oxycedrus* showed strong antibacterial and antifungal activity while aqueous extract of the same plant did not exhibit any sort of antibacterial, antifungal, or anti-candidal effects. This might be due to the nature of extraction solvent used as described previously that methanol is a more powerful solvent for extraction of bioactive constituents especially antimicrobial constituents from medicinal plants as compared to other solvents including water.

## Conclusion

The results obtained in the present study conclude that all the methanol derived fractions of *P. jacquemontiana* leaf extract (except aqueous fraction) have strong antibacterial potential, especially against all MDR strains tested and three fractions (PJM, PJE and PJB) have strong antifungal potential with minimal MIC values. This can attribute to the phytochemical composition of the extracts. They can be a source of novel metabolites. The extracts also proved their anti-candidal activity in these assays and can be utilized further for the discovery of novel anti-candidal agents. Moreover the plant extracts showing outstanding antimicrobial activities can be helpful in exposing novel antibiotic classes that can function as selective agents for health maintenance around the globe.

## Abbreviations

MBC: Minimum bactericidal concentration
MDR: Multi-drug resistant
MFC: Minimum fungicidal concentration
MIC: Minimum inhibitory concentration
PJA: *Parrotiopsis jacquemontiana* soluble residual aqueous fraction of PJM
PJB: *Parrotiopsis jacquemontiana* butanol fraction of PJM
PJC: *Parrotiopsis jacquemontiana* chloroform fraction of ANM
PJE: *Parrotiopsis jacquemontiana* ethyl acetate fraction of PJM
PJH: *Parrotiopsis jacquemontiana* hexane fraction of PJM
PJM: *Parrotiopsis jacquemontiana* methanol extract of leaves
